# Epigenetic Regulation of *BDNF* Gene during Development and Diseases

**DOI:** 10.3390/ijms18030571

**Published:** 2017-03-06

**Authors:** Kuan-Wei Chen, Linyi Chen

**Affiliations:** 1 Institute of Molecular Medicine and Department of Medical Science, National Tsing Hua University, 101, Section 2, Kuang-Fu Road, Hsinchu 30013, Taiwan; dreadlordpop@hotmail.com; 2 Department of Medical Science, National Tsing Hua University, Hsinchu 30013, Taiwan

**Keywords:** BDNF, histone modification, neuronal development, neurodegenerative disease

## Abstract

Brain-derived neurotrophic factor (BDNF) is required for the development of the nervous system, proper cognitive function and memory formation. While aberrant expression of BDNF has been implicated in neurological disorders, the transcriptional regulation of *BDNF* remains to be elucidated. In response to different stimuli, *BDNF* expression can be initiated from different promoters. Several studies have suggested that the expression of *BDNF* is regulated by promoter methylation. An emerging theme points to the possibility that histone modifications at the *BDNF* promoters may link to the neurological pathology. Thus, understanding the epigenetic regulation at the *BDNF* promoters will shed light on future therapies for neurological disorders. The present review summarizes the current knowledge of histone modifications of the *BDNF* gene in neuronal diseases, as well as the developmental regulation of the *BDNF* gene based on data from the Encyclopedia of DNA Elements (ENCODE).

## 1. Introduction

Brain-derived neurotrophic factor (BDNF) belongs to the neurotrophin family and is known to be critical for the development of the brain, neuronal regeneration and synaptic plasticity [1,2,3,4,5,6,7,8,9]. BDNF also participates in long-term potentiation (LTP), and learning and memory [10,11,12,13]. Aberrant levels of BDNF have been implicated in a number of neurological diseases such as Alzheimer’s disease (AD), Parkinson’s disease (PD), Huntington’s disease (HD) and amyotrophic lateral sclerosis (ALS) [14]. Furthermore, the addition of a BDNF protein or elevation of BDNF by the *Bdnf* gene (human gene: *BDNF*; mouse gene: *Bndf*) delivery in animal models of neurological and psychiatric disorders improves memory formation and the survival of neuronal cells [14]. In this review, we summarize the epigenetic regulation of *Bdnf* during neuronal development, focusing on histone modifications based on the data in the Encyclopedia of DNA Elements (ENCODE) database. We also discuss the known regulation of the mouse *Bdnf* gene in diseases.

## 2. *Bdnf* Gene

**Figure 1:**
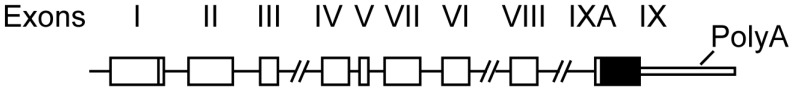
Brain-derived neurotrophic factor (*Bdnf*) gene structure. The mouse *Bdnf* gene is depicted here. White boxes indicate untranslated exons and the black box indicates a coding exon (IX).

Both human and rodent *BDNF* genes contain nine exons (I–IX) (Figure 1) and each exon has its own promoter, resulting in more than 10 different transcripts in both humans and rodents. Interestingly, all transcripts are translated into an identical BDNF protein [15,16,17,18]. Exons I, II, IV and VI of *BDNF* between humans and rodents are highly conserved [19]. Each exon is regulated by its own unique promoter, conferring temporal and spatial control of *Bdnf* expression in an activity-dependent manner. For example, pilocarpine treatment, a muscarinic acetylcholine receptor (mAChR) agonist, stimulates transcription of exons II, IV and VI of *Bdnf* in the neurite, whereas it promotes the transcription of exons V, VII and VIII of *Bdnf* in the soma of the CA1 region within the hippocampus [20]. More studies are needed to understand the complexity of the transcriptional regulation of the *Bdnf* gene.

## 3. Data-Mining Using ENCODE

ENCODE is an open database funded by the National Human Genome Research Institute in the USA [21]. The aim of the ENCODE project is to identify all the regulatory regions of genes and the expression of genes, including gene expression (RNA sequencing), transcription factor binding sites (transcription factor chromatin immunoprecipitation followed by deep sequencing (TF ChIP-seq), chromatin conformations (DNase-seq, chromatin interaction analysis by paired-end tag sequencing (ChIA-PET) and Hi-C), histone modifications (histone ChIP-seq) and RNA immunoprecipitation followed by deep sequencing (RIP-seq). In the ENCODE database, many whole genome sequencing data are uploaded and mapped to the updated human or mouse genome. The ENCODE database provides a user-friendly platform that allows readers to visualize data using the University of California Santa Cruz (UCSC, Santa Cruz, CA, USA) genome browser and making comparisons among different experiments [22]. We used this database to describe gene expression data as well as to compare histone modification results around the *Bdnf* gene based on RNA-sequencing (RNA-seq) data and chromatin immunoprecipitation-deep sequencing (ChIP-seq) results from Dr. Bing Ren’s lab (Department of Cellular and Molecular Medicine, San Diego, CA, USA). In the histone modification ChIP-seq data, the reads were filtered and further processed to peak calling, and additionally normalized to a control to generate a fold change over the control. In the RNA-seq data, the raw data were processed to alignment with the mouse genome. All methods used are available in each track of the ENCODE database. In addition, a similar project, PsychENCODE, was initiated in 2015 [23] and aims to decode the gene expressions and their regulatory domain, as does as ENCODE project, but focuses on human brain diseases. Chromatin states such as DNase-free regions, histone modifications and DNA methylation are decoded in healthy controls and disease-affected neuronal cells and further mapped to the human genome, allowing researchers to analyze this data. Up-to-date, high-throughput data of autism spectrum disorder, schizophrenia and bipolar disorder are available in the PsychENCODE project which can be further investigated by a specific gene as done in this review.

## 4. Histone Modifications in Gene Regulation

Temporal and spatial expression of developmental genes requires precision and plasticity for cell fate determination. Epigenetic regulation can modulate gene expression effectively without changing DNA sequences. The mechanisms of epigenetics include DNA methylation, histone modifications and control by non-coding RNAs. Here, we focus on histone modifications. Eukaryotic DNA wraps around eight core histones (two H2A-H2B dimers, and a H3-H4 tetramer) to form a nucleosome structure [24]. These histones are post-translationally modified by methylation, acetylation, phosphorylation, ubiquitination, sumoylation and other modifications [25]. These modifications alter the affinity between histones and DNA, recruit transcriptional activators/repressors, and in turn regulate gene expression. Histone H3 lysine 4 mono-methylation (H3K4me1) and histone H3 lysine 27 acetylation (H3K27ac) mark the active enhancer region of genes leading to the increase of gene expression [26,27]. On one hand, the combination of H3K4me1 and histone H3 lysine 27 tri-methylation (H3K27me3) indicates a poised enhancer region [28]. The active promoters of genes are marked with histone H3 lysine 4 tri-methylation (H3K4me3) and H3K27ac. In contrast, H3K27me3 indicates a repressive promoter whereas histone H3 lysine 36 tri-methylation (H3K36me3) marks the gene body in general, which can indicate either active or repressive transcription [29,30]. A summary of histone modifications and their functional associations are shown in Table 1.

## 5. Histone Modifications around Mouse *Bdnf*

### 5.1. Histone Modifications around Mouse Bdnf in Embryonic Stem Cells 

**Figure 2:**
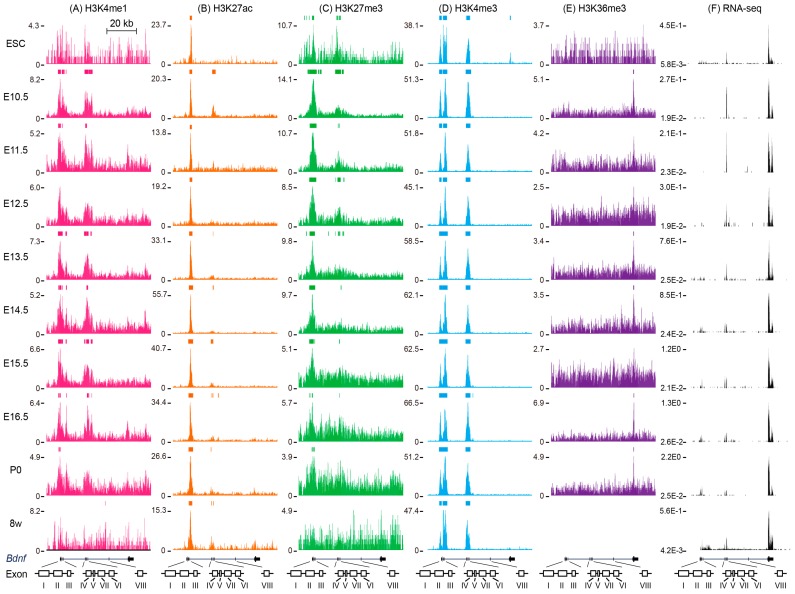
Histone modifications at mouse *Bdnf* gene during brain development. All data were collected from the ENCODE database and visualized in the University of California Santa Cruz (UCSC) genome browser with the alignment to mouse genome assembly (GRCm38/mm10). The bars located above the peaks indicate the confident enrichments of these histone marks. ESC: embryonic stem cell; E10.5–E16.5: embryonic day 10.5–16.5; P0: postnatal day 0; 8 w: eight weeks adult cerebellum. (**A**) The accession numbers of H3K4me1 mark are ENCSR000CBF, 272GNQ, 450ITF, 157IVC, 253IEG, 037HLB, 449EUZ, 678FIT, 391WSS and 000CAL (ESC, E10.5–E16.5, P0 and 8 w, respectively). (**B**) The accession numbers of H3K27ac mark are ENCSR000CDE, 989LUY, 088UKA, 252ONR, 671NSS, 254AHA, 428GHF, 553IWV, 672ZXY and 000CDC (ESC, E10.5–E16.5, P0 and 8 w, respectively). (**C**) The accession numbers of H3K27me3 mark are ENCSR000CFN, 966TCN, 545BRW, 104PWP, 129OJN, 929GXP, 857GQI, 465TIZ, 340ROY and 000CFN (ESC, E10.5–E16.5, P0 and 8 w, respectively). (**D**) The accession numbers of H3K4me3 mark are ENCSR000CBG, 581EJK, 283RFW, 554TSO, 167ZGV, 203KIB, 486MHP, 637CCT, 427ZJU and 000CAK (ESC, E10.5–E16.5, P0 and 8 w, respectively). (**E**) The accession numbers of H3K36me3 mark are ENCSR000CFO, 747ZXL, 535NVF, 764UIE, 066WUD, 702JYV, 487RAU, 205XBQ and 951UWY (ESC, E10.5–E16.5 and P0, respectively). (**F**) The accession numbers of RNA-seq are ENCSR000CWC, 764OPZ, 307BCA, 908JWT, 792RJV, 343YLB, 557RMA, 367ZPZ, 255SDF and 000BZM (ESC, E10.5–E16.5, P0 and 8 w, respectively).

In ESC, a low signal of *Bdnf* exon IX was detected based on the RNA-seq result (Figure 2F). However, no H3K36me3 mark was enriched in this region, which is consistent with other studies in which H3K36me3 usually marks the active genes [31] (Figure 2E). At this stage, promoters I–VII were marked by H3K4me3 (Figure 2D). H3K27ac only appeared in the 3′ regions of exon I–III and no H3K4me1 signal was detected in these regions (Figure 2A,B). Thus, it is possible that low signals of *Bdnf* exon IX at this stage are due to an inactive promoter. H3K27me3 was distributed throughout exons I–III and IV–VII (Figure 2C). Interestingly, these regions were enriched with both H3K27me3 and H3K4me3, indicating a bivalent chromatin mark (Figure 2C,D) [32]. The bivalent chromatin mark might give the cells the ability to turn on or off rapidly in response to developmental cues. Moreover, accumulation of H3K4me3 was found upstream of exon IX, but disappeared during development (Figure 2D). This finding suggests that the expression of *Bdnf* at this stage is driven by a unique H3K4me3 at the promoter of exon IXA, as well as the combination of H3K4me3 and H3K27ac at the promoter of exons I–VII, which should be further validated. The H3K27me3 at promoters I–III can be regulated by sex determining region Y-box 2 (SOX2) occupancy [33]. In both ES cell-derived and adult hippocampal progenitor cells, knockdown of SOX2 increased the H3K27me3 level as well as the occupancy of its methyltransferase, an enhancer of zeste homolog 2 (EZH2) at *Bdnf* promoter I–III [33].

### 5.2. Histone Modifications around Mouse Bdnf during Brain Development

The expression of *Bdnf* exon IX increased from the embryonic to postnatal stage [15]. RNA-seq data from the ENCODE database revealed that transcripts from exon IX, as well as exons I, II, IV and VI, were detected and the expressions of these transcripts increased throughout the developmental program (Figure 2F). Interestingly, the transcripts from exons I and II elevated from E14.5 whereas exons IV and VI increased starting from E10.5, indicating that different *Bdnf* variants are induced during development. Indeed, several studies have pointed out that expressions of different *BDNF* exons were detected in different brain regions during development for both rodents and humans [15,16,17,18]. For instance, in an adult mouse brain, exon V was highly expressed in the cortex and hippocampus, whereas exon III was detected in all brain regions with similar expression [15]. In addition, exon VI was increased in hippocampal CA3, but not CA1 and dentate gyrus neurons in response to the antidepressant drugs fluoxetine and reboxetine [34]. Furthermore, different stimuli induce *Bdnf* transcripts. Kainic acid, a glutamate analogue, induced transcription of *Bdnf* exons I, IV, V VII, VIII and IXA in rat hippocampus while *N*-methyl-d-aspartate (NMDA) treatment identified *Bdnf* exons II and IV, but not I and III, as fast-reacting exons [15,35]. These data suggest that stimulations might affect histone modifications at different promoters, resulting in different transcripts of exons in distinct brain regions. The gene body mark H3K36me3 was enriched at *Bdnf* exon IX in the E10.5, E13.5, E14.5, E16.5 and P0 stages (Figure 2E). The high level of H3K4me3 remained at the promoter or 3′ regions of all *Bdnf* exons except VIII and IX during E10.5 to P0 and remained so until the adult stage (Figure 2D). However, the level of H3K27me3 decreased at the *Bdnf* promoters I–III and was replaced by H3K4me3 during development (from a break line to a continuous line). The level of H3K27me3 modification gradually diminished from E10.5 to P0 within *Bdnf* promoters I–VII and no enrichment was found at the adult stage (Figure 2C). The decline of H3K27me3 may result in the dissociation of EZH2. It has been reported that upon NMDA stimulation, H3K4me3 at the *Bdnf* promoter increased and serine 28 of H3K27me3 was phosphorylated by p38-mitogen-activated protein kinase (MAPK)/mitogen- and stress-activated protein kinase 1 and 2(Msk1/2), which in turn replaced EZH2 [35]. Simultaneously, *Bdnf* expression increased (RNA-seq) (Figure 2F). This result implies that at the late developmental stage, increased *Bdnf* is a result of decreased repressive histone marks. Nonetheless, the decrease of the active mark also regulates *Bdnf* expression. Consistent with the lower transcripts of *Bdnf* exons II and VI, the lower level of H3K27ac was found at *Bdnf* promoters II and VI in elder mice (20 months) compared with young mice (8 months) [36]. The enhancer mark H3K27ac increased from E10.5 to P0 and was maintained until the adult stage, and was distributed at the 3′ regions of exons I–III, which partially overlapped with the H3K4me3 mark (Figure 2B,D). On the other hand, around exons IV–VII, the H3K27ac mark was found at all stages except E11.5–E12.5 (Figure 2B). Acetylation of H3K27 is mediated by p300 and deacetylation is mediated by histone deacetylase HDACs. Indeed, the inhibition of HDAC in neuronal cells increases *Bdnf* expression. For example, treatment of trichostatin A (TSA), a HDAC class I and II inhibitor, increases histone H3 and H4 acetylation (H3ac and H4ac, respectively) at *Bdnf* promoter I in Neuro-2A cells [37]. The addition of suberoylanilide hydroxamic acid (SAHA), another HDAC class I and II inhibitor, elevates both H3ac and H4ac at *Bdnf* promoters I and IV in cortical neurons [38]. The selective acetylation of H3 and H4 at different *Bdnf* promoters may depend on the cell types. Another enhancer mark, H3K4me1, was reduced throughout the developmental stages (Figure 2A). No enhancer for *Bdnf* has been identified thus far. Here, we noticed that H3K4me1 and H3K27ac marks were found simultaneously around exons IV–VII, indicating a candidate enhancer in this region. The interplay between promoters and enhancers confers another layer of gene control. The physical association between promoter and enhancer was found through a CCCTC-binding factor (CTCF) [39,40]. CTCF ChIP-seq combined with chromosome conformation capture followed by next-generation sequencing (4C-seq) revealed multiple regions around *Bdnf* promoters I–III as well as V–VII contact with *Bdnf* promoter IV [41]. Thus, it is possible that CTCF promotes the physical association between different *BDNF* promoters and enhancer(s) around *Bdnf* exons IV–VII to form a high-order chromatin structure, providing additional regulation of *Bdnf.* Environmental cues such as neurotransmitters trigger activity-dependent transcription in the nervous system, leading to changes of behavior. The regulation of *Bdnf* is also activity-dependent. Depolarization of neurons by KCl increases H3K9ac, but decreases H3K9me2 at *Bdnf* promoter IV [42]. Another report showed that elevation of H3K4me2, H3K14ac and H4ac with concomitant decline of H3K9me2 and HDAC1 was observed in *Bdnf* promoter VI upon KCl stimulation [43]. Although there is a high similarity of promoter regions in the human and mouse *BDNF* genes, it remains possible that the regulation of *BDNF* between human and mouse might be different.

## 6. Epigenetics in Neurological Diseases

The regulation of *BDNF* by DNA methylation and non-coding RNA in neurological diseases has been reviewed in References [44,45]. However, the change of histone modifications that affect *BDNF* expression in neurological diseases is an under-investigated area of research. In the following sections, due to limited references to other neurological diseases, only histone modifications of the *BDNF* gene in Huntington’s disease and Alzheimer’s disease are discussed.

### 6.1. Regulation of BDNF by Histone Modifications in Huntington’s Disease

Huntington’s disease is an inherited neurological disorder that results from the degeneration of brain neurons. The expression of *BDNF* was reduced in the brain of HD patients [46]. Furthermore, the elevation of *Bdnf* in the brain of a HD animal model ameliorated HD-associated symptoms [47,48]. The expression of mutated Huntington’s in vitro and in vivo down-regulates *Bdnf* expression [49]. This reduction of *Bdnf* may be due to the alteration of the chromatin state at its promoters. Indeed, in mouse models and patients of HD, a lower level of H3K4me3 is detected at both human *BDNF* and rodent *Bdnf* promoter II compared to controls [50]. Moreover, Jarid1c, the H3K4me3 demethylase, was dysregulated in the HD mouse model [50]. A change of other histone marks was observed at the *BDNF* promoter. Symmetrical arginine methylation of H2A/H4R3 (H2A/H4R3me2s) mediated by protein arginine methyltransferase 5 (PRMT5) was reduced at the *BDNF* promoter II in the brain of HD patients [51,52]. Although PRMT5-mediated H4R3me2s has been shown to silence gene expression, H4R3me2s ChIP-seq data reveal that H4R3me2s locates to both active and inactive genes in ES cells [53]. Thus, it is possible that the *BDNF* promoter PRMT5 acts as a positive regulator. On the other hand, recruitment of the repressive chromatin remodeler may also suppress the expression of *BDNF* transcripts in HD. For example, RE1-silencing transcription factor/neuron-restrictive silencer factor (REST/NRSF) targets the neuron-restrictive silencer element (NRSE) to repress *BDNF* transcript at promoter II [54]. REST has been reported to recruit HDAC1/2, forming a repressive complex to deacetylate H3K9/K14ac [55,56,57,58]. Thus, in HD (aside from reducing active histone marks), an increase of the repressive chromatin remodeler may also lead to transcriptional repression of *BDNF* transcripts. REST also binds to methyl-CpG binding protein 2 (MeCP2) and the binding of MeCP2 at promoter IV reduces both human *BDNF* and rodent *Bdnf* levels in a DNA methylation–dependent manner [59,60,61]. These reports support the possibility that in HD, a repressive chromatin state at the promoter of *BDNF* may underlie the reduction of *BDNF* compared to a normal individual.

### 6.2. Regulation of Human BDNF by Histone Modifications in Alzheimer’s Disease

Alzheimer’s disease is a neurodegenerative disease that results from the abnormal aggregation of protein in neurons, causing neuronal death. In the brain of AD patients, the reduction of *BDNF* expression was observed in several independent studies [62,63]. However, it is unclear whether and how histone modifications may contribute to the reduction of *BDNF* in AD patients. Infusion of amyloid fibrils, one possible pathogenic protein, into the hippocampus of rats induces HDAC2 expression, as well as HDAC2 occupancy at promoter VI of *Bdnf* and thus represses *Bdnf* expression [64]. In an amyloid precursor protein (APP) knockout mouse model—without APP—accumulation of H4ac, but not H3ac or histone H2B acetylation (H2Bac), was observed at *Bdnf* promoter IV in the prefrontal cortex [65]. The reduction of *BDNF* can be regulated indirectly by other factors. For instance, apolipoprotein E3 (ApoE3), a transporting protein of cholesterol, promotes the nuclear export of HDAC6 and thus decreases the level of HDAC6 at *BDNF* promoters III and IV. Along these lines, an increased level of exon IV was also observed. Furthermore, compared to healthy controls, a higher level of HDAC6 was found in nucleus in AD patients [66].

## 7. Conclusions

Genome-wide analysis of high-throughput data allows the identification of new features of chromatin, which reveal the overwhelming complexity of transcriptional control. The regulation of *Bdnf* by histone modifications shows an example of how the transcription of each exon is regulated by histone modifications and the potential promoter–enhancer relay mechanism through a transcription factor or transcriptional complex. Additional data from the analysis of the higher-order chromatin structure will provide more accurate in vivo regulation of the *Bdnf* gene. 

## Figures and Tables

**Table 1:** Histone modifications and their functional associations.

Modification	Functional Association
H3K4me1	Active enhancer
H3K27ac	Active enhancer and promoter
H3K27me3	Inactive chromatin
H3K4me3	Active promoter
H3K36me3	Active or inactive gene body
